# IgA Serological Response for the Diagnosis of Mycobacterium abscessus Infections in Patients with Cystic Fibrosis

**DOI:** 10.1128/spectrum.00192-22

**Published:** 2022-05-18

**Authors:** Vincent Le Moigne, Anne-Laure Roux, Hélène Mahoudo, Gaëtan Christien, Agnès Ferroni, Oana Dumitrescu, Gérard Lina, Jean-Philippe Bouchara, Patrick Plésiat, Jean-Louis Gaillard, Stéphane Canaan, Geneviève Héry-Arnaud, Jean-Louis Herrmann

**Affiliations:** a Université Paris Saclay, UVSQ, Inserm, Infection et Inflammation, Montigny-le-Bretonneux, France; b AP-HP, GHU Paris Saclay, Hôpital Ambroise Paré, Service de Microbiologie, Boulogne-Billancourt, France; c AP-HP, GHU Paris, Hôpital Necker-Enfants Malades, Service de Microbiologie, Paris, France; d Hospices Civils de Lyon, Hôpital de la Croix Rousse-Centre de Biologie Nord, Institut des Agents Infectieux, Laboratoire de Bactériologie, Lyon, France; e Centre International de Recherche en Infectiologie, INSERM U1111, Université de Lyon, Lyon, France; f UNIV Angers, UNIV Brest, Groupe d'Etude des Interactions Hôte-Pathogène (GEIHP, EA 3142), SFR 4208 ICAT, CHU, Angers, France; g Laboratoire de Bactériologie, CHRU de Besançon, UMR CNRS 6249 Chrono-Environnement, Faculté de Médecine-Pharmacie, Université de Bourgogne Franche-Comté, Besançon, France; h Aix-Marseille Univ, CNRS, LISM, IMM FR3479, Marseille, France; i Département de Bactériologie-Virologie, Hygiène et Parasitologie-Mycologie, Centre Hospitalier Universitaire (CHU) de Brest, Brest, France; j Inserm, EFS, UMR 1078, France; Génétique, Génomique fonctionnelle et Biotechnologies », GGB, Brest, France; k AP-HP, GHU Paris Saclay, Hôpital Raymond Poincaré, Service de Microbiologie, Garches, France; University of Manitoba

**Keywords:** non-tuberculous mycobacteria, cystic fibrosis, IgA, serology, serodiagnosis, ELISA

## Abstract

The immunoglobulin A (IgA) status of cystic fibrosis (CF) patients, presenting with or without a non-tuberculous mycobacterial (NTM) infection, has to date not been fully elucidated toward two antigenic preparations previously described. We have chosen to determine the clinical values of an IgA ELISA for the diagnosis of NTM and/or Mycobacterium abscessus infections in CF patients. One hundred and 73 sera from CF patients, comprising 33 patients with M. abscessus positive cultures, and 31 non-CF healthy controls were assessed. IgA levels were evaluated by indirect ELISAs using a surface antigenic extract named TLR2eF for TLR2 positive extract and a recombinant protein, the phospholipase C (rMAB_0555 or rPLC). These assays revealed a sensitivity of 52.6% (95% CI = 35.8% to 69%) and 42.1% (95% CI = 26.3% to 59.2%) using TLR2eF and rPLC, respectively, and respective specificities of 92.6% (95% CI = 87.5% to 96.1%) and 92% (95% CI = 86.7% to 95.7%) for samples culture positive for M. abscessus. Overall sensitivity and specificity of 66.7% and 85.4%, respectively, were calculated for IgA detection in M. abscessus-culture positive CF patients, when we combine the results of the two used antigens, thus demonstrating the efficiency in detection of positive cases for these two antigens with IgA isotype. CF patients with a positive culture for M. abscessus had the highest IgA titers against TLR2eF and rPLC. The diagnosis of NTM infections, including those due to M. abscessus, can be improved by the addition of an IgA serological assay, especially when cultures, for example, are negative. Based on these promising results, a serological follow-up of a larger number of patients should be performed to determine if the IgA response may be correlated with an active/acute infection state or a very recent infection.

**IMPORTANCE** Mycobacterium abscessus is currently the most frequently isolated rapid growing mycobacterium in human pathology and the major one involved in lung infections. It has recently emerged as responsible for severe pulmonary infections in patients with cystic fibrosis (CF) or those who have undergone lung transplantation. In addition, it represents the most antibiotic resistant mycobacterial species. However, despite its increasing clinical importance, very little is known about the use of M. abscessus parietal compounds and the host response. This has led to the development of serological tests to measure the antibody response in infected patients, and potentially to link this to the culture of respiratory samples. Herein, we describe an important analysis of the serological IgA response from CF patients, and we demonstrate the full diagnostic usefulness of this assay in the diagnosis of NTM infections, and more particularly M. abscessus, in CF patients.

## INTRODUCTION

IgA are antibodies classically produced by mucous membranes, but with regard to mycobacterial infections, much attention has been devoted to the evaluation of specific IgG in most serological studies. However, IgA antibodies have been shown to be useful in the diagnosis of various infections due to fungi (Candida albicans, *Trichosporon cutaneum*) or parasites (*Leishmania*, *Toxoplasma*), as well as viruses, including SARS-CoV-2 (1, 2) and bacteria. Among bacterial infections, the IgA response has been investigated in infections caused by Bordetella pertussis (3), Chlamydia trachomatis, and mycobacteria, and especially Mycobacterium tuberculosis, with about three quarters of all IgA studies concerning this pathogen within the Mycobacterium genus (4–8). Other mycobacterial infections targeted in IgA serological studies, especially in cystic fibrosis (CF) patients, were Mycobacterium avium (9) or M. abscessus (10, 11).

An enzyme immunoassay kit developed by the group of Kitada (12, 13) was used by Jeong et al. (10) to detect IgA antibodies reacting to a glycopeptidolipid (GPL) core antigen derived from M. avium complex. With this reagent, they were able to detect patients infected with M. abscessus or Mycobacterium massiliense without differentiating them from other mycobacteria belonging to the avium complex, whereas they differentiated those with M. tuberculosis, linked to the fact that GPLs exist only in non-tuberculous mycobacterials (NTMs). The same reagent (Tauns Laboratories) was used in three recent studies in 2020 (14–16) to identify patients infected with M. abscessus. Although, this assay did not allow the differentiation of sera from patients infected with M. abscessus complex mycobacteria or with the M. avium species complex (MAC), it predicted a favorable outcome, revealing a decrease over time of antibody level in surgically treated patients (15). Decrease of GPL core IgA antibodies in patient sera was also correlated with antibiotic treatment efficacy (15), indicative of the usefulness of IgA serum level to predict the recurrence of disease or treatment efficacy.

As part of a diagnostic accuracy study, we demonstrate the importance of IgG antibodies detection for the diagnosis of NTM infections in CF patients, and more accurately for those infected by M. abscessus infections with two antigenic preparations (17). Using the same serum collection and the same antigens, we evaluated the potential of the IgA detection technique in enhancing the diagnosis of NTM infections, in order to decipher a more complete humoral response in CF patients infected by NTM, and to see if the IgA response might be a new diagnostic tool for detecting NTM-infected CF patients, and more specifically M. abscessus*-*infected CF patients.

## RESULTS

**FIG 1:**
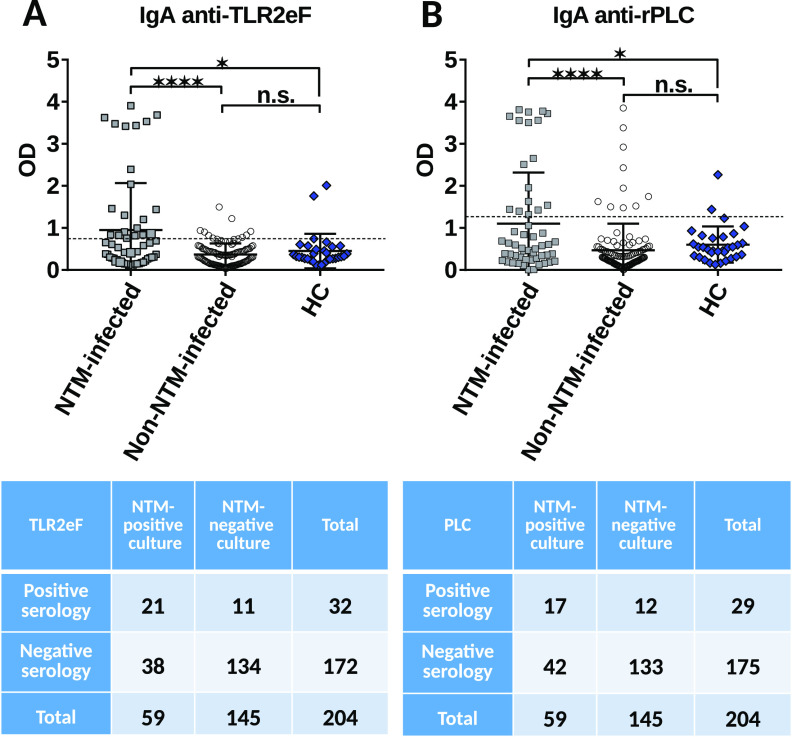
IgA response of NTM positive culture, non-NTM positive culture groups and healthy control (HC) group using TLR2eF (A) and rPLC (B) as antigens. Each dot represents one patient in the scattergrams. Horizontal lines represent the mean and vertical bars SDs. Values are presented in Tables S1A and S1B in the supplemental material for each antigenic sample, respectively. Chosen cut-off values (test positivity threshold) are respectively 0.751 (A) and 1.274 (B) (dotted horizontal lines). *P < *0.001 for comparisons of NTM-culture positive groups *versus* the non-NTM group plus the HC group for TLR2eF and rPLC. Beneath each figure is represented the corresponding 2 by 2 table for NTM-culture positive patients. n.s., nonsignificant; *, *P <* 0.05; **, *P <* 0.01; ***, *P <* 0.001; ****, *P <* 0.0001.

We first evaluated the diagnostic value of the recombinant PLC (rPLC) and surface-extract TLR2 activators-enriched fraction (TLR2eF) IgA ELISAs by analyzing the sera of 59 CF patients NTM-infected to the sera of 114 CF patients NTM-non-infected or a control group (31 healthy controls [HC] non-CF). The IgA antibody titers against TLR2eF were significantly different between both CF-groups (*P < *0.0001) (Fig. 1A). The area under the receiver operating characteristic (ROC) curve (AUC) was 0.645, allowing the determination of a cut-off value of 0.751 (Fig. S1A). This values allowed us to obtain specificity and sensitivity of 92.5% (95% CI = 86.9% to 96.2%) and 38.2% (95% CI = 25.4% to 52.3%), respectively, for the TLR2eF ELISA for detecting NTM culture-positive individuals.

Similarly, IgA antibody titers against rPLC distinctly differentiated both groups (*P < *0.0001) (Fig. 1B). The area under the ROC curve (AUC) was 0.655, allowing the determination of a cut-off value of 1.274 (Fig. S1B). This value allowed us to obtain specificity and sensitivity of 91.8% (95% CI = 86.1% to 95.7%) and 30.9% (95% CI = 19.1% to 44.8%), respectively, specifically for the detection of NTM-culture positive-CF patients with the rPLC ELISA slightly lower than those obtained with TLR2eF. Positive and negative predictive values (PPV and NPV), positive and negative likelihood ratios (PLR and NLR), and accuracy were calculated based on a prevalence of 3.6% according to the results obtained previously (18). We provide the values for both antigens in Tables S1A and S1B. The most interesting score was obtained with TLR2eF as antigenic extract.

**FIG 2:**
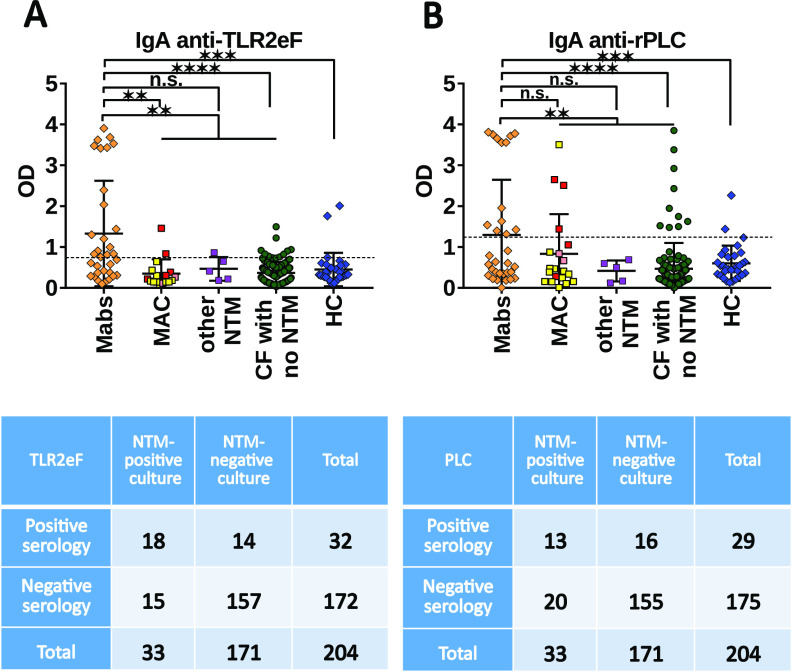
IgA response of the different cystic fibrosis (CF) patient groups and healthy control (HC) group using TLR2eF (A) or rPLC (B) as antigen. Each dot represents one patient in the scattergrams. In MAC group, yellow squares represent M. avium*-*, pink squares represent *M. chimaera-* and red squares M. intracellulare*-*culture positive-CF patients. Horizontal lines represent the mean and vertical bars SDs. Chosen cut-off values (test positivity threshold) are 0.751 and 1.274 in A and B, respectively (dotted horizontal lines). Beneath each figure is represented the corresponding 2 by 2 table, for Mabs culture positive-CF patients, and see the 2 by 2 Tables S2A and S2B in the supplemental material for MAC culture positive-CF patients. n.s., nonsignificant; *, *P <* 0.05; **, *P <* 0.01; ***, *P <* 0.001; ****, *P <* 0.0001.

Then, with a between mycobacterial species comparison, the TLR2eF antigenic extract allows to obtain Optical density (OD) values that significantly separated CF patients with M. abscessus (Mabs) positive cultures from the other patient groups (*P < *0.01) (Fig. 2A), except for the CF patient group with M. intracellulare (Mint) positive cultures (*P = *0.25), when separated from the group of CF patients with M. avium complex (MAC) positive cultures (Fig. S3B). When Mabs culture positive-CF patients were compared to the other patient groups including the healthy controls, the specificity and sensitivity for the detection of Mabs culture positive-CF patients were 92.6% (95% CI = 87.5% to 96.1%) and 52.6% (95% CI = 35.8% to 69%), respectively. The specificity and sensitivity for detecting MAC culture positive-CF patients were by comparison 92% and 9.5%, respectively (Table S2B).

We also demonstrated distinct and significant differences between CF groups when we used rPLC as antigenic target, with a higher average response for Mabs culture positive-CF patients compared with all other patient groups (*P < *0.01) with the exception of MAC culture positive-CF patients (*P = *0.32) or other NTM culture positive-CF patients (*P = *0.15) (Fig. 2B). The specificity and sensitivity for the detection of Mabs culture positive-CF patients were 92% (95% CI = 86.7% to 95.7%) and 42.1% (95% CI: = 26.3% to 59.2%), respectively. Specificity and sensitivity for detecting MAC culture positive-CF patients were, by comparison, 92% and 19.1%, respectively (Table S2B). Although the differences were not significant, the OD values measured for Mint culture positive-CF patients were higher than those obtained for M. avium plus M. chimaera (Mav/Mchm) culture positive-CF patients (*P = *0.085) and, to a lesser extent, for Mabs culture positive-CF patients (*P = *0.65) (Fig. S3B). PPV and NPV, PLR and NLR, and accuracy for both antigens are given in Tables S3A and S3B. Here again, the most convincing performances were found with TLR2eF antigenic extract.

Finally, we put together results for each antigen (TLR2eF and rPLC), and classified sera as a positive result when at least one of the two assays was positive, and in a negative category when the results were negative for each of the two ELISAs. A comparison of these data with culture results was then achieved (Table 1 and 2). The specificity and sensitivity to detect CF patients with NTM positive cultures, calculated from Table 1, were 85.5% and 44.1%, respectively, and 85.4% and 66.7%, respectively, calculated from Table 2, when focusing on patients with Mabs positive cultures. PPV and NPV, PLR and NLR, and accuracy for these two tables are presented just below their corresponding two by two table from which they were calculated, with a NPV of 98.6% when comparing patients with Mabs positive and Mabs negative cultures.

**TABLE 1:** Two by two table when combining TLR2eF and rPLC serology results versus NTM culture^*a*^

TLR2eF or rPLC	NTM positive culture	NTM negative culture	Total
Positive serology	26	21	47
Negative serology	33	124	157
Total	59	145	204

Parameter	Value	95% CI
PPV (%)	10.2	6.5 to 15.6
NPV (%)	97.6	97.0 to 98.1
PLR	3.0	1.9 to 5.0
NLR	0.65	0.52 to 0.83
Accuracy (%)	84	78.3 to 88.8

a Corresponding values of positive predictive value (PPV), negative predictive value (NPV), positive likelihood ratio (PLR), negative likelihood ratio (NLR), and accuracy and their respective confidence interval at 95% (95% CI).

**TABLE 2:** Two by two table when combining TLR2eF and rPLC serology results versus Mabs culture^*a*^

TLR2eF or rPLC	Mabs positive culture	Mabs negative culture	Total
Positive serology	22	25	47
Negative serology	11	146	157
Total	33	171	204

Parameter	Value	95% CI
PPV (%)	14.55	9.9 to 20.8
NPV (%)	98.6	97.7 to 99.1
PLR	4.6	2.95 to 7.05
NLR	0.39	0.24 to 0.64
Accuracy (%)	84.7	79.0 to 89.3

a Corresponding values of positive predictive value (PPV), negative predictive value (NPV), positive likelihood ratio (PLR), negative likelihood ratio (NLR), and accuracy and their respective confidence interval at 95% (95% CI).

## DISCUSSION

The importance of the IgA response against mycobacterial pathogens has been demonstrated in several studies (6–10, 19). Both mucosal and systemic IgA have protective effects and can trigger pro-inflammatory response (20, 21). Furthermore, with a half-life four time shorter than IgG, serum specific IgA might reflect a recent active infection. For these reasons, the evaluation of IgA, alongside IgG measurement, makes sense. In addition, the combination of IgA and IgG responses may help to improve serodiagnosis test accuracy for active NTM and/or M. abscessus infection.

In this study, framed as an exploration of the suitability of the serum IgA assay for the diagnosis of NTM infections, we have shown low sensitivity values when each antigen was evaluated separately, around 30% and up to 50% when considering Mabs-positive CF-patients; but with a doubling in sensitivity when the two antigens were combined, culminating in a value of 60%, specifically for the diagnosis of Mabs infections. These results confirm that the use of a cocktail of antigens instead of single antigens increases the sensitivity of mycobacterial serodiagnosis. This might be due to the differential expression of certain antigens during the development of mycobacteria (22). In addition, this multi-antigen analysis gives even better results when IgG and IgA levels are cumulated, like improving sensitivity from 23% to 62% as described by Juliàn et al. (23) for M. tuberculosis. Introducing the IgG results obtained from the same cohort (17) allowed us to see that when at least three results are positive, we obtained a sensitivity of 60.6%, a specificity of 95.3% and positive and negative predictive values of 32.6% and 98.5%, respectively (Table S4). Similarly, these sensitivity values can reach 90% if at least one of the tests is positive i.e., either IgA with TLR2e, or IgA with rPLC and likewise IgG (Table S4). Our study has shown that this enzyme immunoassay measuring the IgA response against M. abscessus rPLC and the TLR2eF extract might well complement the IgG data and help to distinguish M. abscessus from M. avium pulmonary infections as well as other infections or lung diseases. Finally and most interestingly, in all cases the negative predictive value is greater than or equal to 97.5%, making a strong argument in the setting of a negative IgG and or IgA response for excluding NTM infection in CF patients. In the context of pharmacological regimes which restore CFTR, or with patients with non-productive cough, such a result might avoid additional investigations or even unnecessary treatment in these patients.

Unlike the only test measuring IgA to detect Mabs infection published to date (10, 14–16), our IgA ELISA was able to differentiate Mabs from Mav/Mchm-infections, but not from Mint-infections although the number of patients was small. Our assay is based on the recognition of Mabs proteins or extracts while the older test is based on detection of antibodies recognizing the GPL core antigen of M. avium.

Both ELISAs are currently being tested in a prospective study to determine the prevalence of NTM infections in CF patients (clinical trial no. ID RCB: 2017-A00025-48). In addition, variations in IgA levels over time might be indicative of disease development in the setting of rising levels, or in the case of a decrease, might provide proof of successful antibiotic treatment as it has been shown with TB infection (8). This is also an area of interest which we are studying in this cohort, in addition to the comparative IgA/IgG kinetics.

## MATERIALS AND METHODS

### Patients and antigens.

Serum samples for antibody determination, antigens, or preparations used in this study as well as the distribution of the different groups depending on the results of sputum cultures are the same as those previously described (17).

### ELISAs.

ELISAs were developed as described previously (17). First, the coating of plates was completed overnight at 4°C with 1 μg/mL of each antigen, recombinant PLC (rPLC), or the surface-extract TLR2 activators-enriched fraction (TLR2eF) diluted in 100 μL of carbonate-bicarbonate buffer (0.1 M, pH 9.6). Then, plates were washed with phosphate-buffered saline (PBS) twice and blocked by incubation for 1 h at 37°C with 200 μL of PBS containing 1% bovine serum albumin (PBS-BSA). Sera were diluted at a 1/400th dilution in PBS-Tween 20 (0.05% vol/vol) (PBS-T) containing 0.5% BSA (PBS-T-BSA) and added to the plates. Plates were incubated for 90 min at 37°C and, following four washes with PBS-T, alkaline phosphatase-conjugated goat anti-human IgA (Southern biotechnology, Birmingham, USA) diluted in PBS-T-BSA was added. A second incubation of 90 min of the plates was performed at 37°C and four additional washes with PBS-T are realized before the addition of alkaline phosphatase substrate, 100 μL of 1 mg/mL of *p*-nitrophenylphosphate (Sigma, Saint Quentin Fallavier, France) diluted in diethanolamine buffer (pH 9.8). Plates were finally incubated in the dark at room temperature for 120 min. Ultimately, the spectrophotometric reading of the plates was determined at the wavelength of 405 nm. All serum were tested in duplicate. OD mean ± standard deviation was calculated for each patient group. The test positivity thresholds, sensitivities, and specificities of the ELISAs were assessed by calculating the AUC ROC curve. ROC curves were generated using GraphPad Prism 6.0 software (GraphPad Software, La Jolla, CA, USA). The closest point to the upper left side corner was used to determine the cut-off value. ROC curves corresponding to Fig. 1 and 2 are presented in Fig. S1 and S2 in the supplemental material.

### Data analysis.

A Student’s *t* test was used for all analyses using GraphPad Prism 6.0 software (GraphPad Software, La Jolla, CA, USA). A two-sided *P* of < 0.05 was considered to be statistically significant.
