# Supplementary material for: IgA Serological Response for the Diagnosis of Mycobacterium abscessus Infections in Patients with Cystic Fibrosis
Source: Microbiol Spectr. 2022 May 18;10(3):e00192-22. doi: 10.1128/spectrum.00192-22 (PMC9241595; doi:10.1128/spectrum.00192-22)
Supplement: SUPPLEMENTAL FILE 1 — Supplemental material. Download spectrum.00192-22-s0001.pdf, PDF file, 0.7 MB [file spectrum.00192-22-s0001.pdf]

Supplementary Tables S1A and S1B : positive predictive values (PPV), negative predictive values (NPV), positive likelihood ratio (PLR), negative likelihood ratio (NLR) and accuracy and their respective confidence interval at 95% (95% CI) of results presented in Fig. 1.

A

TLR2eF		95% CI
PPV (%)	14.9	8.3 to 25.4
NPV (%)	97.5	96.9 to 97.9
PLR	4.7	2.4 to 9.1
NLR	0.7	0.57 to 0.85
Accuracy (%)	90.3	85.5 to 94.0

B

rPLC		95% CI
PPV (%)	11.5	6.2 to 20.3
NPV (%)	97.2	96.7 to 97.6
PLR	3.5	1.77 to 6.83
NLR	0.78	0.66 to 0.92
Accuracy (%)	89.5	84.4 to 93.3

Supplementary Figures S1A and S1B

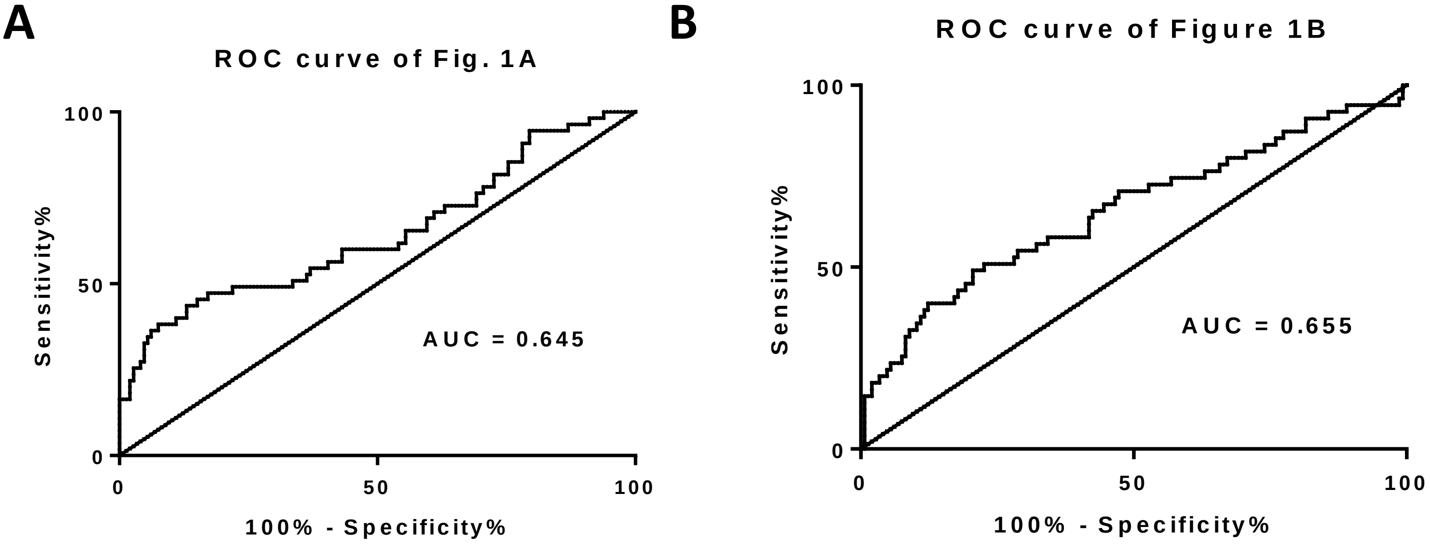

Supplementary Figure S1A and S1B: Sensitivity of the enzyme immunoassays presented in Fig. 1 between NTM-infected and Non-NTM-infected groups plus the control group for the enzyme immunoassays against: (A) the surface-extract TLR2 activators-enriched fraction and (B) PLC recombinant protein.

Receiver operating characteristic (ROC) curve for detection of IgA anti-TLR2eF (A) and anti-rPLC (B) have respectively an area under the curve [AUC] of 0.645 and 0.655.

Supplementary Tables S2A and S2B: Two by two tables showing serology results compared to MAC-positive or negative cultures, using the different antigens: A: TLR2eF ; B: rPLC. Patients with Mabs-positive culture were excluded.

A

TLR2eF	MAC-positive culture	MAC-negative culture	Total
Positive serology	2	12	14
Negative serology	19	138	157
Total	21	150	171

B

rPLC	MAC-positive culture	MAC-negative culture	Total
Positive serology	4	12	16
Negative serology	17	138	155
Total	21	150	171

Supplementary Figures S2A and S2B

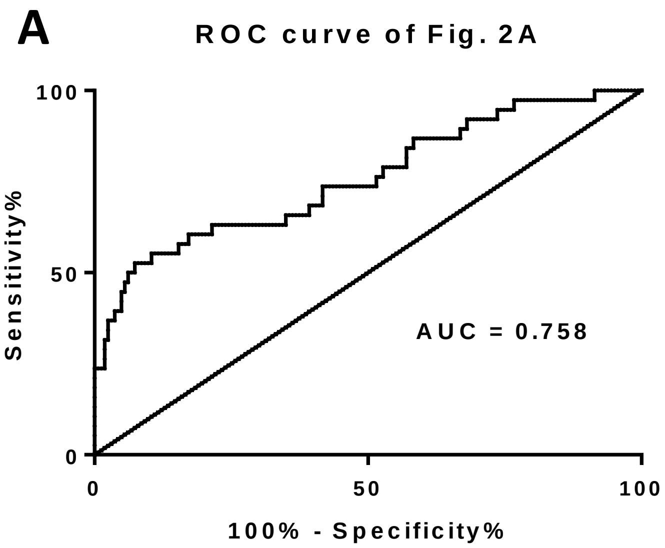

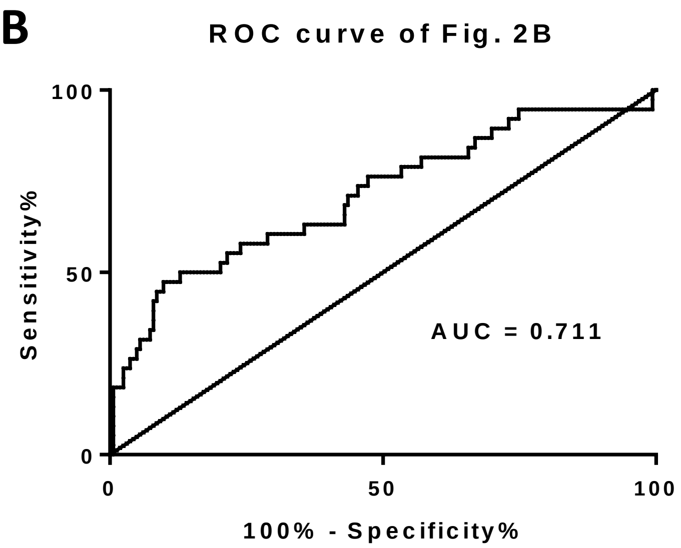

Supplementary Figure S2A and S2B: Sensitivity of the enzyme immunoassays presented in Fig. 2 between Mabs-infected plus Mint-infected groups and other CF patients infected groups plus control group for the enzyme immunoassays against: (A) the surface-extract TLR2 activators-enriched fraction and (B) PLC recombinant protein. Receiver operating characteristic (ROC) curve for detection of IgG anti- TLR2eF (A), and anti-rPLC (B) have respectively an area under the curve [AUC] of 0.758 and 0.711.

Supplementary Figures S3A and S3B

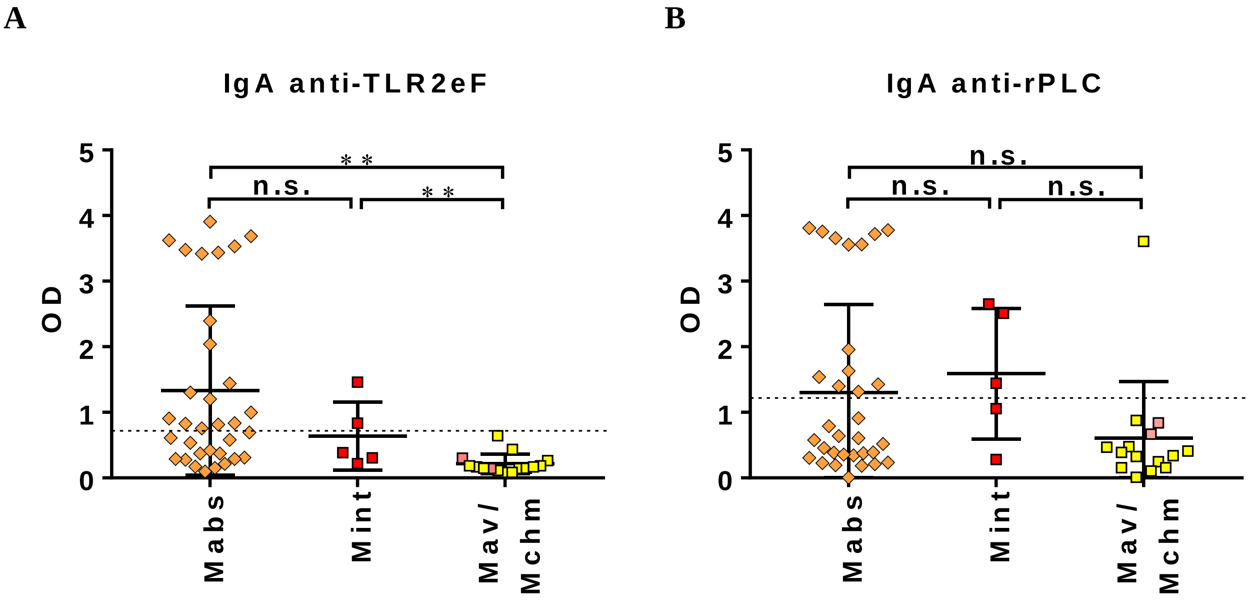

Supplementary Figure S3A and S3B: IgA response of the different cystic fibrosis (CF) patient groups with positive cultures for *M. abscessus* (Mabs), *M. intracellulare* (Mint) or *M. avium* plus *M. chimaera* (Mav/Mchm) opposite to: (A) TLR2eF or the surface-extract TLR2 activating-enriched fraction and and (B) rPLC. Each dot represents one patient in the scattergrams. Yellow squares represent *M. avium*- and pink squares represent *M. chimaera*-positive cultures in the Mav/Mchm group. Horizontal lines represent the mean and vertical bars SDs.

Supplementary Tables S3A and S3B : positive predictive values (PPV), negative predictive values (NPV), positive likelihood ratio (PLR), negative likelihood ratio (NLR) and accuracy and their respective confidence interval at 95% (95% CI) of results presented in Supplementary Figure 3.

A

TLR2eF		95% CI
PPV (%)	19.9	12.1 to 31.0
NPV (%)	98.2	97.4 to 98.75
PLR	6.6	3.7 to 12.0
NLR	0.5	0.34 to 0.72
Accuracy (%)	90.47	85.6 to 94.1

B

rPLC		95% CI
PPV (%)	13.6	7.7 to 22.8
NPV (%)	97.6	96.8 to 98.1
PLR	4.2	2.2 to 7.9
NLR	0.67	0.51 to 0.88
Accuracy (%)	88.8	83.6 to 92.8

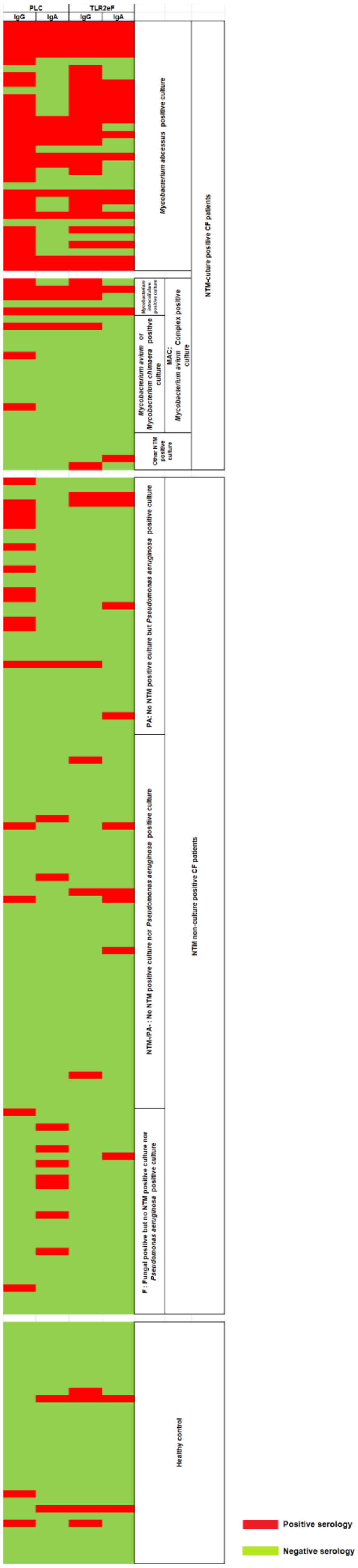

Supplementary Figure S4: **IgA and IgG responses compilation**. Results previously obtained for IgG antibodies (1) were taken back and added to IgA results: a heat-map/matrix was designed with results obtained for each individual sera with both antigenic preparation, PLC and TLR2eF, and for both serotype. If a sera is above the calculated threshold, this positive result induce a red square. A negative result is represented by a green square. Groups designed according bacterial culture positivity or not are represented on the right.

Supplementary Table S4: IgA and IgG responses compilation. Results previously obtained for IgG antibodies (1) were taken back and added to IgA results: a heat-map/matrix was designed with results obtained for each individual sera with both antigenic preparation, PLC and TLR2eF, and for both serotype (**Fig. S4** in the supplemental material). The resulting Table allow to see the number of case in different groups if at least one test is positive, if at least two tests, at least three tests or if the four tests are positive. Corresponding values of sensitivity, specificity, positive predictive value (PPV), negative predictive value (NPV), positive likelihood ratio (PLR), negative likelihood ratio (NLR) and accuracy and their respective confidence interval at 95% (95% CI) are indicated.

	**Postitive test number**				
	**0**	**At least 1**	**At least 2**	**At least 3**	**4**
**Mabs positive culture**	**2**	**31**	**24**	**20**	**11**
M. intracellulare	**1**	**4**	**4**	**3**	**2**
**MAC (without *M. intacellulare*)**	**13**	**3**	**1**	**1**	**0**
**Other NTM**	**3**	**2**	**0**	**0**	**0**
**Non NTM + HC**	**108**	**37**	**8**	**4**	**0**
**Total**	**127**	**77**	**37**	**28**	**13**

		**Value** (95% CI)			
**Sensitivity**		**93.9%**<br>(79.8% to 99.3%)	**72.7%**<br>(54.5% to 86.7%)	**60.6%**<br>(42.1% to 77.1%)	**33.3%**<br>(17.9% to 51.8%)
**Specificity**		**73.1%**<br>(65.8% to 79.6%)	**92.4%**<br>(87.3% to 95.9%)	**95.3%**<br>(91.0% to 98.0%)	**98.8%**<br>(95.8% to 99.9%)
**PPV**		**11.5%**<br>(9.1% to 14.5%)	**26.3%**<br>(16.9% to 38.5%)	**32.6%**<br>(18.9% to 50.1%)	**51.6%**<br>(19.8% to 82.1%)
**NPV**		**99.7%**<br>(98.8% to 99.9%)	**98.9%**<br>(98.1% to 99.4%)	**98.5%**<br>(97.7% to 99.0%)	**97.5%**<br>(96.9% to 98.1%)
**Accuracy**		**73.8%**<br>(67.2% to 79.7%)	**91.7%**<br>(87.0% to 95.1%)	**94.1%**<br>(89.9% to 96.9%)	**96.5%**<br>(92.9% to 98.5%)
**PLR**		**3.5**<br>(2.7 to 4.5)	**9.6**<br>(5.4 to 16.8)	**12.9**<br>(6.2 to 26.9)	**28.5**<br>(6.6 to 122.7)
**NLR**		**0.08**<br>(0.02 to 0.32)	**0.30**<br>(0.17 to 0.52)	**0.41**<br>(0.27 to 0.63)	**0.67**<br>(0.53 to 0.86)

REFERENCES

1. Le Moigne V, Roux AL, Mahoudo H, Christien G, Ferroni A, Dumitrescu O, Lina G, Bouchara JP, Plésiat P, Gaillard JL, Canaan S, Héry-Arnaud G, Herrmann JL. 2021. Serological biomarkers for the diagnosis of *Mycobacterium abscessus* infections in cystic fibrosis patients. *J Cyst Fibros* 9:S1569-1993(21)01360-6. doi: 10.1016/j.jcf.2021.08.019.
